# Tocotrienol-Enriched Beverage Enhances Psychological Well-Being, Antioxidant Defense, and Genomic Stability in Older Adults: A Randomized Controlled Trial

**DOI:** 10.3390/nu17132179

**Published:** 2025-06-30

**Authors:** Razinah Sharif, Mah Kit Wai, Ooi Theng Choon, Sitti Rahma Abdul Hafid, Tze Yan Lee

**Affiliations:** 1 Centre for Healthy Ageing and Wellness, Faculty of Health Sciences, Universiti Kebangsaan Malaysia, Jalan Raja Muda Abdul Aziz, Kuala Lumpur 50300, Malaysia; 2 Premier Integrated Labs Sdn. Bhd., Kuala Lumpur 55100, Malaysia; thengchoon.ooi@premierintegratedlabs.com.my; 3 Malaysian Palm Oil Board, Persiaran Institusi, Bandar Baru Bangi, Kajang 43000, Malaysia; ctrahma@mpob.gov.my; 4 Clinical Laboratory Science Section, Institute of Medical Science Technology, Universiti Kuala Lumpur, Kajang 43000, Malaysia

**Keywords:** tocotrienol, vitamin E, older adults, telomerase, psychological well-being, oxidative stress, randomized controlled trial

## Abstract

**Background:** This study investigates the effects of a tocotrienol-enriched drink on oxidative damage and genomic stability in older adults over a 6-month period. **Methods:** A total of 67 participants (27 males and 40 females, mean age 60.45 ± 5.75 years) were enrolled in this double-blinded, two-arm, parallel randomized controlled trial. Baseline, mid-point, and end-point assessments were conducted to monitor various health parameters. Significant Group × Time interaction effects were observed for several key outcomes. **Results:** Group A demonstrated significantly better improvements in QOL-Psychological (*p* = 0.014, Partial Eta Squared = 0.153), suggesting a beneficial impact of tocotrienol supplementation on mental well-being. Additionally, Group A showed more favorable trends in TNF-α (*p* = 0.04), T-SOD (*p* = 0.04), catalase (*p* = 0.02), and telomerase (*p* = 0.02), suggesting potential antioxidant and genomic stability improvements over time. **Conclusions:** In a nutshell, tocotrienol supplementation may exert beneficial effects on psychological well-being, oxidative stress modulation, and genomic stability in aging populations.

## 1. Introduction

Aging is a multifactorial biological process characterized by the progressive decline in physiological integrity, leading to increased susceptibility to chronic diseases, cognitive decline, and frailty. Several interconnected mechanisms contribute to this process, including oxidative stress, low-grade chronic inflammation, and genomic instability [1]. Oxidative stress results from an imbalance between reactive oxygen species (ROS) production and the antioxidant defense system, causing cumulative damage to lipids, proteins, and DNA [2]. At the same time, chronic inflammation—or “inflammaging”—manifests as elevated levels of circulating pro-inflammatory cytokines, which can impair tissue repair and promote degenerative diseases [3]. Together, these factors accelerate cellular senescence, mitochondrial dysfunction, and the decline in systemic resilience.

Among the many biomarkers implicated in aging, telomere length and telomerase activity have emerged as key indicators of cellular aging. Telomeres, repetitive DNA sequences at the ends of chromosomes, shorten with each cell division. Excessive telomere attrition leads to genomic instability and senescence. Oxidative stress is one of the primary accelerators of telomere shortening [4]. Telomerase, an enzyme capable of extending telomeres, is typically downregulated in most somatic cells with age. Interventions that modulate oxidative stress and inflammation may therefore have the potential to preserve telomere length and delay cellular aging [5].

One promising class of compounds for combating the hallmarks of aging is vitamin E, particularly tocotrienols—a less studied but more potent form compared to the widely known tocopherols. Both belong to the vitamin E family, consisting of four tocopherol and four tocotrienol isoforms (α, β, γ, δ). Unlike tocopherols, tocotrienols have unsaturated isoprenoid side chains, which allow for more efficient incorporation into cellular membranes and better tissue penetration [6]. Tocotrienols have demonstrated potent antioxidant, anti-inflammatory, neuroprotective, and cholesterol-lowering effects in vitro and in vivo [7]. Importantly, their biological activity is not redundant to that of tocopherols; in fact, tocopherols can sometimes blunt the effects of tocotrienols when co-administered [8]. Tocotrienols are found in palm oil, rice bran, barley, and annatto seeds. Their mechanisms of action include the suppression of NF-κB–mediated inflammatory cytokine production and the activation of the Nrf2-ARE pathway, enhancing the expression of antioxidant enzymes like SOD and catalase [6,9].

Several preclinical and human studies have shown that tocotrienols modulate molecular pathways relevant to aging. In one randomized controlled trial, tocotrienol-rich fraction (TRF) supplementation in healthy older adults improved oxidative status and lipid profiles without adverse effects [10]. Another study reported that tocotrienols reduced white matter lesions and preserved brain function in aging individuals [11].

Despite these encouraging findings, only a paucity of well-controlled clinical trials has comprehensively assessed the role of tocotrienols in modulating the multi-system biomarkers of aging in older adults. Most existing studies are limited in duration, focus on specific outcomes like cholesterol or inflammation, and rarely assess markers of genomic integrity such as telomerase or 8-OHdG. Moreover, a few trials have evaluated tocotrienol’s effects in Southeast Asian populations, despite the regional abundance of palm-derived tocotrienol-rich fractions and their inclusion in local diets. Therefore, a targeted intervention using a tocotrienol-enriched functional drink may yield meaningful translational insights, particularly in an aging Malaysian population.

In light of all these gaps, this randomized controlled trial aimed to evaluate the effects of a tocotrienol-enriched beverage on a multidimensional panel of aging-related biomarkers in community-dwelling older adults. The study investigated changes in inflammatory cytokines, antioxidant enzyme activity, telomerase levels, oxidative DNA damage, and psychological well-being over a 6-month intervention period. It was hypothesized that daily tocotrienol supplementation would yield measurable improvements in these markers compared to placebo. The findings from this trial provide a more comprehensive view of tocotrienol’s potential to promote healthy aging at the molecular and systemic levels.

## 2. Materials and Methods

### 2.1. Study Design and Ethical Approval

This study was conducted as a double-blinded, two-arm, parallel-group, randomized controlled trial (RCT) over a 6-month period. The research was approved by the Universiti Kebangsaan Malaysia (UKM) Research Ethics Committee under the reference number JEP-2024-966. All participants provided written informed consent before enrollment. The study adhered to the ethical principles outlined in the Declaration of Helsinki and complied with Good Clinical Practice (GCP) standards.

### 2.2. Participants and Randomization

A total of 67 participants were recruited from community health screenings and outreach programs in urban areas of Malaysia. A priori power calculation based on α = 0.05, power = 0.8, and an expected moderate effect size (f = 0.25) suggested a minimum of 60 participants. The final sample of 67 ensured sufficient power for primary endpoints. Demographics closely reflected urban aging populations in Malaysia. Eligible participants were aged 50 to 70 years and in general good health, with no diagnosis of chronic inflammatory, autoimmune, hepatic, renal, or malignancy-related conditions. Individuals on long-term antioxidant supplements or participating in other intervention trials were excluded to minimize confounding factors. Additional exclusion criteria included uncontrolled diabetes, hypertension, or recent surgery. A computer-generated randomization list was used to assign participants to either the intervention group (Group A) or placebo group (Group B) in a 1:1 ratio. Allocation was concealed using sealed, opaque envelopes. Both investigators and participants were blinded to group assignments throughout the trial.

### 2.3. Investigational Product

The intervention involved a functional beverage formulated with PhytoGaia’s TocoGaia–a tocotrienol-rich fraction (TRF) derived from palm oil. Each serving contained approximately 200 mg of mixed tocotrienols, predominantly γ- and δ-isomers, which are known for their superior antioxidant and anti-inflammatory properties as reported in Table 1. The beverage was formulated and packaged under Good Manufacturing Practice (GMP) conditions. The placebo beverage, identical in taste, color, and packaging, did not contain any tocotrienols. Participants in both groups were instructed to consume one serving per day, preferably in the morning after breakfast, for a continuous period of 6 months. Adherence was monitored through monthly follow-ups and by collecting unused product containers.

### 2.4. Outcome Measures and Data Collection

Outcomes were assessed at three time points: baseline (week 0), mid-point (week 12), and end-point (week 24). Demographic and anthropometric data were collected using standardized forms. Height, weight, waist and hip circumference, and body mass index (BMI) were measured following WHO protocols. Clinical history and comorbidity profiles were assessed via structured interviews. Blood pressure was recorded in a seated position using an automated sphygmomanometer after 5 min of rest.

Psychological well-being and quality of life were measured using the validated WHOQOL-BREF questionnaire, which includes physical, psychological, social, and environmental domains. Cognitive function was evaluated using the Mini-Mental State Examination (MMSE), while physical performance was assessed using the Timed-Up-and-Go (TUG) test and handgrip strength (measured with a Jamar digital dynamometer). These assessments were administered by trained personnel following standardized procedures.

### 2.5. Blood Samples and Biomarker Testing

Fasting venous blood samples were collected by certified phlebotomists at all three time points. Samples were centrifuged within one hour of collection, and plasma and serum were aliquoted and stored at −80 °C until analysis. Hematological parameters and biochemical profiles—including complete blood count, liver enzymes (ALT, AST, GGT), renal function (urea, creatinine, uric acid), lipid profile (TC, LDL, HDL, TG), and fasting glucose—were analyzed using automated clinical analyzers in an ISO-certified laboratory.

Inflammatory biomarkers measured included TNF-α, IL-6, IL-10, IL-1β, IL-8, and TGF-β, while oxidative stress markers included MDA, total superoxide dismutase (T-SOD), total glutathione (T-GSH), total antioxidant capacity (T-AOC), and catalase. Other measured biomarkers included cortisol, insulin, telomerase activity, and 8-hydroxy-2-deoxyguanosine (8-OHdG). All assays were conducted using commercial enzyme-linked immunosorbent assay (ELISA) kits validated for human serum or plasma. All tests were run in duplicate, and intra-assay coefficients of variation were maintained below 10%.

### 2.6. Statistical Analysis

Data analysis was performed using IBM SPSS Statistics version 27. All variables were assessed for normality using the Shapiro–Wilk test. Continuous variables were presented as mean ± standard deviation (SD), and categorical variables were reported as frequencies and percentages. Between-group differences at baseline were tested using independent sample *t*-tests (for continuous data) and chi-square tests (for categorical data). The primary analysis involved repeated measures analysis of variance (ANOVA) to assess time, group, and interaction (Group × Time) effects. Where interaction effects were significant, post-hoc tests were conducted using Bonferroni correction. Effect sizes were reported using partial eta squared (η^2^), with 0.01, 0.06, and 0.14 interpreted as small, medium, and large effects, respectively. A *p*-value < 0.05 was considered statistically significant.

## 3. Results

### 3.1. Baseline Characteristics

**Figure 1:**
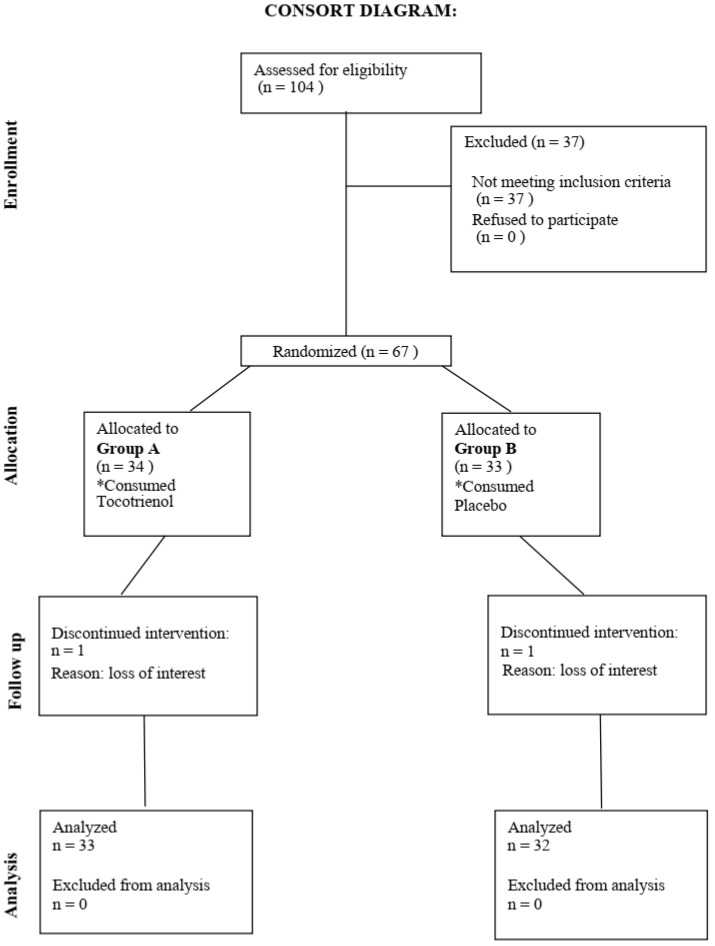
Study CONSORT flow diagram. Consort diagram of patients eligible, recruited, numbers followed up, and included in analysis.

Figure 1 showed study CONSORT flow diagram. Consort diagram of patients eligible, recruited, numbers followed up, and included in analysis were reported in this figure. A total of 65 participants finished the 6 months intervention.

At baseline, both intervention and placebo groups were statistically comparable in terms of demographic, anthropometric, and clinical characteristics. The average age across the cohort was 60.45 ± 5.75 years, with a gender distribution of 27 males and 40 females. The mean body mass index (BMI) was within the normal-to-overweight range, and no significant differences in waist-hip ratio or blood pressure were observed. Cognitive and physical function indicators—including MMSE scores, Timed-Up-and-Go (TUG) test duration, and handgrip strength—were also similar between groups. Likewise, baseline blood parameters, including full blood count, liver and renal profiles, and fasting glucose levels, did not differ significantly between the two groups.

This suggests successful randomization and baseline equivalence, minimizing potential bias and allowing valid longitudinal comparisons between intervention and control groups. Table 2 summarizes the key baseline variables.

### 3.2. Inflammatory, Antioxidant, and Other Biomarker Changes

Overall, the tocotrienol group exhibited a broader pattern of systemic anti-inflammatory effect. These findings are depicted in Table 3. As shown in Table 4, significant differences between Group A and Group B were observed for several biomarkers. For IL-6, Group A demonstrated higher mean levels (2.53 ± 3.21 pg/mL) compared to Group B (1.97 ± 2.89 pg/mL), with a *p*-value of 0.03 and a Partial Eta Squared value of 0.12, indicating a medium effect size. Similarly, for MDA, Group A had higher mean levels (1310.23 ± 110.15 ng/mL) compared to Group B (1256.14 ± 120.17 ng/mL), with a *p*-value of 0.02 and a Partial Eta Squared value of 0.14, suggesting a moderate effect size.

For TGF-beta, Group B had slightly higher mean levels (80.12 ± 14.98 pg/mL) compared to Group A (74.8 ± 15.23 pg/mL), with a *p*-value of 0.04 and a Partial Eta Squared value of 0.09, reflecting a small-to-moderate effect. Additionally, catalase levels were significantly higher in Group A (20.45 ± 5.21 U/mL) than in Group B (19.32 ± 4.85 U/mL), with a *p*-value of 0.04 and a Partial Eta Squared value of 0.12, suggesting a moderate effect size. For insulin, group B showed higher levels (7.92 ± 2.81 uIU/mL) compared to Group A (6.75 ± 3.15 uIU/mL), with a *p*-value of 0.01 and a Partial Eta Squared value of 0.15, indicating a strong effect size.

Time significantly influenced multiple biomarkers. For IL-6, there was a significant time effect with a *p*-value of 0.001 and a Partial Eta Squared value of 0.20, indicating a large effect size. Additionally, for MDA, mean values decreased significantly over time with a *p*-value of 0.02 and a Partial Eta Squared value of 0.13, suggesting a moderate effect size. Similarly, TGF-beta displayed a significant time effect with a *p*-value of 0.03 and a Partial Eta Squared value of 0.11, indicating a small-to-moderate effect size.

Cortisol also showed a significant time effect, decreasing over time with a *p*-value of 0.01 and a Partial Eta Squared value of 0.15, reflecting a moderate-to-large effect size. Lastly, for OHdG, time had a significant impact (*p*-value = 0.04) with a Partial Eta Squared value of 0.11, indicating a small-to-moderate effect size.

Significant interaction effects were observed for several biomarkers. For TNF-a, the interaction effect was significant (*p*-value = 0.04) with a Partial Eta Squared value of 0.12, suggesting a moderate effect size. Similarly, for T-SOD, there was a significant interaction (*p*-value = 0.04) with a Partial Eta Squared value of 0.12, indicating a moderate effect size.

For catalase, a significant interaction effect was noted (*p*-value = 0.02) with a Partial Eta Squared value of 0.10, suggesting a moderate effect size. Lastly, for telomerase, there was a significant interaction effect (*p*-value = 0.02) with a Partial Eta Squared value of 0.10, suggesting a moderate effect size.

Overall, significant group differences were observed primarily for IL-6, MDA, TGF-beta, catalase, and insulin, with Group A generally showing higher values in inflammatory and antioxidant biomarkers. Time effects were prominent in IL-6, MDA, TGF-beta, cortisol, and OHdG, indicating meaningful trends across the study’s timeline. Interaction effects further highlight distinct responses to interventions or environmental factors for TNF-a, T-SOD, catalase, and telomerase. These findings underscore the potential influences of group membership and time on these physiological and biochemical parameters.

## 4. Discussion

This study demonstrated that daily tocotrienol supplementation over six months can positively influence several key markers of biological aging in older adults, including psychological well-being, inflammatory cytokines, antioxidant enzyme activity, and telomerase levels. These findings are in line with the evolving body of evidence that supports the use of tocotrienols as multi-targeted bioactive compounds for healthy aging. The randomized, double-blind, placebo-controlled design strengthens the internal validity of these results, while the inclusion of multidimensional outcomes reflects the complex interplay of oxidative, inflammatory, and genomic factors in aging physiology.

The most pronounced improvement observed was in the psychological domain of quality of life, where a significant Group × Time interaction favored the tocotrienol group. This aligns with findings by Gopalan and his colleagues, who reported improved cognitive and emotional health following tocotrienol supplementation in aging adults [11]. Chronic inflammation and oxidative stress have been implicated in mood disorders and cognitive impairment due to their impact on neurotransmitter metabolism, neuroinflammation, and hippocampal plasticity [12]. By lowering inflammatory burden and boosting endogenous antioxidant defenses, tocotrienols may indirectly enhance mental health and emotional resilience.

In the domain of inflammatory modulation, our results revealed a significant reduction in TNF-α and downward trends for other cytokines such as IL-6 and TGF-β in the intervention group. Tocotrienols have previously been shown to suppress the NF-κB signaling pathway, a master regulator of pro-inflammatory gene expression [9]. In a clinical trial by Qureshi et al., TRF supplementation led to significant reductions in inflammatory biomarkers among hyperlipidemic patients [13]. This study supports those findings in an older population, suggesting that tocotrienols could mitigate “inflammaging”, the chronic low-grade inflammation associated with aging and frailty [3].

Antioxidant enzyme activity also improved significantly with tocotrienol supplementation, particularly T-SOD and catalase levels. These enzymes form the first line of defense against reactive oxygen species, converting superoxide radicals and hydrogen peroxide into less harmful molecules. Prior studies have demonstrated that tocotrienols can activate the Nrf2 pathway, upregulating these antioxidant systems [6]. The modest reduction in MDA, a marker of lipid peroxidation, further supports the oxidative stress-lowering effects of tocotrienols. Chin and colleagues observed similar antioxidant effects in a randomized trial in older Malaysians, reinforcing the relevance of these findings to the local population [10].

Another key outcome was the significant increase in telomerase activity in the tocotrienol group. Telomerase is a ribonucleoprotein enzyme that maintains telomere length and is critical for genomic stability. Shortened telomeres have been associated with oxidative stress, chronic inflammation, and elevated risk of age-related diseases [5,14,15]. By reducing oxidative DNA damage, tocotrienols may help preserve telomere length and delay cellular senescence. Although few human trials have examined this effect, various preclinical data support tocotrienol’s role in modulating telomere biology [8,16,17,18].

The lack of significant change in cognitive test scores or physical performance (MMSE, handgrip strength, and TUG test) may reflect the relatively short duration of the intervention and the high baseline function of participants. Nonetheless, the maintenance of performance—paired with significant improvements in biological markers—suggests a stabilizing effect. Similar findings have been reported in preventive trials where biomarker changes precede overt clinical improvements [19]. It is also possible that longer-term supplementation or targeting populations with lower baseline function would yield more substantial effects.

More importantly, no adverse effects or safety concerns were observed throughout the study. Blood biochemistry remained within reference ranges, including red blood cells and platelet counts, which were monitored as safety parameters. This confirms previous safety data on tocotrienol-rich fractions, particularly palm-derived TRF, which has been shown to be well tolerated in both healthy and clinical populations [7]. Taken together, these findings support the potential of tocotrienol as a safe and effective nutraceutical intervention for mitigating age-associated biological changes.

While this study provides valuable insights into the potential benefits of tocotrienol supplementation in aging populations, several limitations should be acknowledged. First, while the tocotrienol-rich beverage used was derived from a standardized commercial formulation with known tocotrienol content, in-house testing of antioxidant potential (e.g., ORAC, DPPH) was not conducted, which is a limitation in fully characterizing the product’s functional properties. However, the percentages of vitamin E, as measured via HPLC, were included for more detailed characterization. The investigational product contained mixed tocotrienol as the main ingredient. The sample size, although sufficient for detecting moderate effect sizes in biomarker outcomes, may have limited the statistical power to detect smaller effects, particularly in physical and cognitive performance measures. A larger cohort would enhance the generalizability and robustness of the findings.

Second, the duration of the intervention was limited to six months, which, while longer than many previous tocotrienol trials, may still be insufficient to observe significant changes in slowly progressing physiological domains such as muscle strength or cognitive function. Longer-term studies are warranted to validate these preliminary findings.

Third, the reliance on self-reported questionnaires for psychological well-being, while validated, introduces a degree of subjectivity and potential bias. Future studies may consider including objective clinical assessments or neuroimaging biomarkers to strengthen the interpretation of psychological effects. The study did not include urine biomarker analysis or advanced body composition techniques such as DEXA. Future trials could incorporate these to provide more comprehensive metabolic and nutritional insights.

Additionally, dietary intake and lifestyle factors such as physical activity were not strictly controlled or monitored during the trial. Although participants were advised to maintain their usual habits, these variables could act as confounders. A more rigorous assessment of diet and lifestyle would allow for a clearer attribution of effects to the intervention. The influence of dietary patterns on antioxidant and inflammatory biomarkers cannot be excluded.

Lastly, although compliance was monitored, the study did not include serum tocotrienol level measurements to biochemically confirm adherence. Incorporating such biomarkers in future trials would strengthen dose-response interpretations and intervention fidelity.

Despite these limitations, this trial contributes significantly to the clinical understanding of tocotrienol’s multifaceted role in modulating aging-related biomarkers, providing a strong foundation for larger and longer-term investigations.

## 5. Conclusions

This randomized controlled trial demonstrated that daily supplementation with a tocotrienol-enriched beverage over a 6-month period led to significant improvements in psychological well-being, antioxidant enzyme activity, and telomerase levels among older adults. These effects were accompanied by favorable trends in inflammatory and oxidative stress markers, supporting the hypothesis that tocotrienols modulate multiple hallmarks of aging at the molecular and systemic level. While no significant changes were observed in cognitive or physical performance, the maintenance of function alongside improved biomarker profiles suggests a stabilizing and preventive effect. The intervention was well tolerated with no adverse events, reinforcing the safety of tocotrienol supplementation in this population. This trial provides preliminary evidence supporting tocotrienol’s potential in promoting healthy aging through biomarker modulation. While findings are promising, larger and longer trials are needed to confirm these effects and assess clinical relevance.

## Figures and Tables

**Table 1:** Percentage of vitamin E in investigational product.

Total Vitamin E (Tocopherols and Tocotrienols)	TocoGaia (P)–Natural Full Spectrum Tocotrienols/Tocopherol Complex Powder
d-alpha-Tocopherol	8.0%
Total d-Mixed-Tocotrienol	23%
d-alpha-Tocotrienol	8.0%
d-Beta-Tocotrienol	0.3%
d-Gamma-Tocotrienol	9.5%
d-Delta-Tocotrienol	5.0%

**Table 2:** Baseline characteristics of participants.

Variables	Group A (Tocotrienol)	Group B (Placebo)
Age (years)	60.4 ± 5.7	60.5 ± 5.8
Sex (M/F)	13/20	14/19
BMI (kg/m^2^)	24.8 ± 3.1	25.1 ± 3.4
MMSE Score	27.1 ± 1.6	27.0 ± 1.8
TUG (s)	9.8 ± 1.2	9.9 ± 1.3
Handgrip (kg)	24.5 ± 4.3	24.2 ± 4.5

Values are presented as mean ± standard deviation (SD) for continuous variables and frequency (n) for categorical variables. No statistically significant differences were observed between Group A (Tocotrienol) and Group B (Placebo) at baseline (*p* > 0.05 for all comparisons), indicating successful randomization. A *p*-value < 0.05 was considered statistically significant. Groups were randomized, and any baseline differences are assumed to be due to chance.

**Table 3:** Mean ± SD values for biomarkers at baseline, mid-point, and end-point for Group A and Group B.

Marker	Baseline (Group A Mean ± SD)	Baseline (Group B Mean ± SD)	Mid-Point (Group A Mean ± SD)	Mid-Point (Group B Mean ± SD)	End-Point (Group A Mean ± SD)	End-Point (Group B Mean ± SD)
IL-6 (pg/mL)	2.85 ± 3.25	2.65 ± 3.15	2.40 ± 3.00	1.85 ± 2.80	1.99 ± 2.70	1.75 ± 2.65
IL-10 (pg/mL)	1.75 ± 2.65	2.10 ± 2.85	1.62 ± 2.48	1.95 ± 2.70	1.45 ± 2.38	1.72 ± 2.50
IL-1beta (pg/mL)	2.35 ± 2.32	2.55 ± 2.50	2.10 ± 2.07	2.32 ± 2.27	1.88 ± 2.10	2.01 ± 2.14
IL-8 (pg/mL)	1280.22 ± 215.67	1320.00 ± 208.23	1215.45 ± 202.32	1260.12 ± 194.78	1120.90 ± 188.45	1170.80 ± 192.21
TNF-a (pg/mL)	111.35 ± 21.55	107.12 ± 21.04	106.25 ± 20.67	105.45 ± 19.90	103.45 ± 19.55	101.35 ± 18.76
TGF-beta (pg/mL)	75.92 ± 15.34	80.10 ± 16.12	72.78 ± 14.11	78.89 ± 15.56	70.89 ± 13.23	74.56 ± 13.85
MDA (ng/mL)	1340.56 ± 125.34	1301.34 ± 118.90	1275.10 ± 113.20	1239.80 ± 109.87	1195.11 ± 110.12	1175.67 ± 106.54
T-SOD (U/mL)	108.65 ± 18.23	101.45 ± 17.90	99.12 ± 16.20	97.22 ± 16.54	93.56 ± 15.25	90.12 ± 14.78
T-GSH (umol/L)	5.97 ± 1.12	5.79 ± 1.25	5.32 ± 1.15	5.15 ± 1.05	4.85 ± 1.02	4.65 ± 0.98
T-AOC (μmol/mL)	482.45 ± 55.32	468.10 ± 52.81	455.89 ± 50.34	445.30 ± 49.12	440.45 ± 48.50	430.12 ± 47.05
Catalase (U/mL)	22.14 ± 5.54	20.45 ± 4.98	19.05 ± 4.78	17.90 ± 4.45	18.50 ± 4.30	15.90 ± 3.98
Cortisol (ng/mL)	210.32 ± 33.12	200.54 ± 32.05	195.76 ± 30.23	185.12 ± 28.56	185.98 ± 28.10	175.45 ± 27.67
Insulin (uIU/mL)	8.15 ± 3.21	7.65 ± 2.98	7.01 ± 3.10	6.49 ± 2.85	6.32 ± 2.78	6.05 ± 2.64
Telomerase (ng/mL)	15.89 ± 5.13	15.36 ± 4.87	14.12 ± 4.34	13.90 ± 4.56	13.25 ± 4.11	12.87 ± 3.95
OHdG (ng/mL)	72.36 ± 11.87	77.89 ± 10.21	69.45 ± 9.98	72.56 ± 10.12	66.45 ± 9.56	70.22 ± 10.54

**Table 4:** Statistical analysis of inflammatory, antioxidant, and other biomarkers across groups and time points.

Marker	Group Effect (*p*-Value)	Group Effect (Partial Eta Sq)	Time Effect (*p*-Value)	Time Effect (Partial Eta Sq)	Group × Time Interaction (*p*-Value)	Group × Time Interaction (Partial Eta Sq)
Inflammatory Markers						
IL-6 (pg/mL)	0.03 *	0.12	0.001 *	0.20	0.15	0.08
IL-10 (pg/mL)	0.45	0.02	0.14	0.07	0.41	0.03
IL-1beta (pg/mL)	0.51	0.01	0.17	0.05	0.31	0.04
IL-8 (pg/mL)	0.10	0.04	0.09	0.06	0.19	0.05
TNF-a (pg/mL)	0.19	0.05	0.18	0.07	0.04 *	0.12
TGF-beta (pg/mL)	0.04 *	0.09	0.03	0.11	0.18	0.07
Antioxidants						
MDA (ng/mL)	0.02 *	0.14	0.02 *	0.13	0.25	0.06
T-SOD (U/mL)	0.27	0.03	0.09	0.08	0.04 *	0.12
T-GSH (umol/L)	0.10	0.08	0.07	0.05	0.06	0.08
T-AOC (μmol/mL)	0.25	0.06	0.11	0.07	0.17	0.06
Catalase (U/mL)	0.04 *	0.12	0.09	0.10	0.02 *	0.10
Others						
Cortisol (ng/mL)	0.30	0.02	0.01 *	0.15	0.19	0.04
Insulin (uIU/mL)	0.01 *	0.15	0.10	0.12	0.22	0.05
Telomerase (ng/mL)	0.22	0.04	0.06	0.09	0.02 *	0.10
OHdG (ng/mL)	0.44	0.03	0.04 *	0.11	0.17	0.06
Vitamin E (μg/mL)	0.57	0.02	0.34	0.03	0.62	0.01

* Values are presented as mean ± SD. *p* < 0.05 is considered statistically significant. Significant effects are reported for Group Effect (differences between Group A and Group B), Time Effect (changes across baseline, mid-point, and end-point), and Group × Time Interaction Effect (interaction between group and time factors).

## Data Availability

The original contributions presented in this study are included in the article. Further inquiries can be directed to the corresponding authors.
